# Evaluating Pneumonitis Incidence in Patients with Non–small Cell Lung Cancer Treated with Immunotherapy and/or Chemotherapy Using Real-world and Clinical Trial Data

**DOI:** 10.1158/2767-9764.CRC-22-0370

**Published:** 2023-02-14

**Authors:** Qi Liu, Chenan Zhang, Yue Huang, Ruihao Huang, Shiew-Mei Huang, Erin Larkins, Liza Stapleford, Donna R. Rivera, Paul G. Kluetz, Shenggang Wang, Hao Zhu, James Weese, Elizabeth Cromartie, Mahder Teka, Sheetal Walters, Frank Wolf, Thomas D. Brown

**Affiliations:** 1 Office of Clinical Pharmacology, FDA, Silver Spring, Maryland.; 2 Syapse, San Francisco, California.; 3 Office of Oncologic Diseases, FDA, Silver Spring, Maryland.; 4 Oncology Center of Excellence, FDA, Silver Spring, Maryland.; 5 Advocate Aurora Health, Milwaukee, Wisconsin.

## Abstract

Pneumonitis is a potentially life-threatening complication of anticancer therapy, and future treatment decisions may be informed by characterizing patients receiving therapies in the real-world setting. In this study, the incidence of treatment-associated pneumonitis (TAP) was compared among patients with advanced non–small cell lung cancer receiving immune checkpoint inhibitors (ICI) or chemotherapies in either of two settings: randomized clinical trials (RCT) or real world data (RWD)-based clinical practice. Pneumonitis cases were identified using International Classification of Diseases codes (for RWD), or the Medical Dictionary for Regulatory Activities preferred terms (for RCTs). TAP was defined as pneumonitis diagnosed during treatment or within 30 days of the last treatment administration. Overall TAP rates in the RWD cohort were lower [ICI: 1.9%; 95% confidence interval (CI), 1.2–3.2; chemotherapy: 0.8%; 95% CI, 0.4–1.6] than overall rates in the RCT cohort (ICI: 5.6%; 95% CI, 5.0–6.2; chemotherapy: 1.2%; 95% CI, 0.9–1.5). Overall RWD TAP rates were similar to grade 3+ RCT TAP rates (ICI: 2.0%; 95% CI, 1.6–2.3; chemotherapy: 0.6%; 95% CI, 0.4–0.9). In both cohorts, higher TAP incidence was observed among patients with a past medical history of pneumonitis than those without, regardless of treatment group. On the basis of this sizable study leveraging RWD, TAP incidence was low in the RWD cohort, likely in part due to methodology used for RWD focusing on clinically significant cases. Past medical history of pneumonitis was associated with TAP in both cohorts.

**Significance:**

Pneumonitis is a potentially life-threatening complication of anticancer treatment. As treatment options expand, management decisions become increasingly complex, and there is a greater need to understand the safety profiles of the treatment options in the real-world setting. Real-world data serve as an additional source of valuable information to complement clinical trial data and inform understanding of toxicity in patients with non–small cell lung cancer receiving ICIs or chemotherapies.

## Introduction

The non–small cell lung cancer (NSCLC) treatment landscape shifted in 2015 when the FDA approved the first immune checkpoint inhibitors (ICI) for treatment of NSCLC (1). With six ICIs (atezolizumab, cemiplimab, durvalumab, ipilimumab, nivolumab, pembrolizumab) approved in the United States for treatment of advanced NSCLC, clinicians have various immunotherapies to choose from for use as monotherapy or in combination therapy. As treatment options expand, management decisions become increasingly complex, and there is a greater need to understand the safety profiles of the treatment options in order to inform personalized assessments of risk-benefit ratios for individual patients.

ICIs downregulate pathways that suppress T-cell activation to enhance antitumor immune responses in patients with cancer, but this upshift in immune-mediated activity can also result in “off-target” immune-related adverse drug events, including pneumonitis (2, 3). Thoracic radiotherapy has been linked to higher rates of treatment-related pneumonitis in patients treated with chemotherapy as well as ICIs (4–7), and both clinical and dosimetric factors appear to influence the actual level of risk in treatment-related pneumonitis in patients treated with chemoradiotherapy (8, 9). Although evidence of significant association is limited to date, other risk factors for treatment-related pneumonitis have been assessed in meta-analyses of trial data and in real-world NSCLC studies, including age, histology, prior interstitial lung disease, and other chronic pulmonary diseases (10–12).

Clinical and radiographic characteristics of ICI-related pneumonitis include cough and dyspnea as well as ground glass opacities on chest CT scans (13, 14). On the basis of literature data, clinical trials tend to report lower pneumonitis rates among ICI-treated patients (3%–5%; refs. 15–18) compared with real-world studies (4%–19%; refs. 11, 12, 19). Prior data from clinical trials and expanded access programs typically describe higher rates of pneumonitis for ICI therapy compared with chemotherapy, although comparisons vary with respect to tumor type, specific ICI agents assessed, type of regimen (monotherapy, multiagent or multimodality regimen), and prior treatment (16, 19, 20). While ICI-related fatal toxic events occur uncommonly and at a comparable frequency with other oncologic interventions, they do occur at a rate of 0.3% to 1.3%, with the types of fatal adverse events differing markedly between regimens (21).

Given the limited, and sometimes conflicting estimates in the current literature, this study was conducted to address two objectives: (i) to describe the occurrence of treatment-associated pneumonitis (TAP) in patients with NSCLC in the real-world data (RWD) setting compared with randomized clinical trials (RCT; treated with either ICI or chemotherapy); and (ii) to describe potential risk factors of patients diagnosed with TAP.

## Materials and Methods

### Data Source

Data from patients treated in routine clinical practice (RWD) and patients who participated in RCTs were analyzed separately.

#### RWD Cohort

This was a retrospective observational cohort study of patients who were diagnosed with locally advanced or metastatic NSCLC (aNSCLC) and were treated at a community health system in the Midwestern United States comprised of more than 25 hospitals and over 500 health care delivery sites between January 1, 2010 and September 14, 2020. Eligibility for the cohort included stage III or IV disease at diagnosis or progression to metastatic disease prior to treatment with either ICI (monotherapy or in combination with chemotherapy) or chemotherapy (without concomitant radiation). Although pneumonitis has been associated with EGFR-TKI (tyrosine kinase inhibitor) therapies (22), such therapies were not assessed in this study. Data for the analysis were obtained from a combination of structured databases with technology-enabled data curation and certified cancer registrar (CTR) abstraction. For suspected pneumonitis cases identified with structured data, additional targeted patient chart review was conducted by a team of clinical oncologists and CTRs for verification of pneumonitis diagnosis and treatment, as well as collection of additional clinical information. The targeted chart review included assessment of oncology notes, nursing notes, treatment summaries, and other unstructured information sources captured in the electronic health records of patients in the study cohort. Data elements assessed in the targeted chart review included: confirmation of clinical characteristics, treatment information, unique pneumonitis diagnosis, and details on prior thoracic radiotherapy.

### Variables

Patients were classified into two treatment groups. The ICI group included patients with ICI treatment (atezolizumab, nivolumab, or pembrolizumab) initiated after their advanced and/or metastatic diagnosis. Durvalumab was excluded because of its sole initial approved indication as consolidation therapy following concurrent chemoradiation in patients with locally advanced disease. Cemiplimab was excluded because of the recency of its approval for NSCLC. The chemotherapy group included patients with chemotherapy-containing regimens initiated after their advanced and/or metastatic diagnosis. Patients in the ICI group with a prior history of chemotherapy treatment were eligible for inclusion, while patients in the chemotherapy group were excluded if they had a history of ICI therapy. The intent of this exclusion is to restrict the comparison with those patients with a first exposure to ICI and those patients with no prior exposure to ICI. Patients receiving ICI or chemotherapy as consolidation therapy following definitive thoracic radiation were not indexed at the time of consolidation therapy but were indexed at treatment initiation for progressive disease (the date the therapy was received). Index treatment dates for either treatment groups were defined as the first date of an observed treatment administration of ICI or chemotherapy in the advanced and/or metastatic setting. This corresponds to when a therapy was first administered with palliative rather than definitive and/or curative intent, to align with core eligibility criteria from clinical trials. The chemotherapy group was limited to patients who received their initial chemotherapy treatment on or after January 1, 2010, as radiation practice patterns and technological changes emerging around that time period may be associated with reduced incidence of high-grade pneumonitis after definitive chemoradiation. Additional sensitivity analyses were also performed restricting the analysis to patients who received their initial chemotherapy treatment on or after January 1, 2015 to further assess comparability with the ICI treatment group.

Patients with pneumonitis diagnoses were initially identified from the structured database by screening for relevant International Classification of Diseases (ICD) codes. Pneumonitis was defined using terms (including subclasses) in ICD-9 and ICD-10 that included “pneumonitis” or associated concepts in the Unified Medical Language System (Supplementary Table S1). Further patient chart review excluded cases of pneumonitis with documented infectious etiology.

TAP was defined as pneumonitis diagnosed after the index treatment start date and within 30 days of the last administration of the index treatment. Pneumonitis cases diagnosed after the 30-day window were considered treatment-associated if clinician notes explicitly attributed the pneumonitis to treatment. Because treatment-related adverse events can occur up to 2 years after treatment cessation (23), sensitivity analyses were performed to assess longer windows for defining TAP, including 60- and 90-day periods. Pneumonitis cases diagnosed prior to index treatment start date, including those that occurred during adjuvant or neoadjuvant treatments, were considered as prior history of pneumonitis, and therefore, not TAP. Cases documented as radiation pneumonitis diagnoses occurring within the TAP attribution window as described above are considered TAP.

### Covariates

Patient demographic and clinical characteristics included: patient sex, race, age at aNSCLC diagnosis, age at index treatment start date, stage at diagnosis, *de novo* versus progressive metastatic disease, histology, smoking status at diagnosis, history of chronic respiratory diseases (Supplementary Table S2), follow-up time, and vital status. As data on thoracic radiotherapy status were not available for all patients in the RWD cohort, a subset of the overall RWD cohort was assessed as a representative sample to establish the prevalence of thoracic radiotherapy prior to index treatment. Radiotherapy data were readily accessible for this subset of patients (*n* = 821) primarily due to linkage to tumor registry data.

For patients in the RWD cohort who were determined to have been diagnosed with pneumonitis at any point in the baseline or follow-up periods, the following additional variables were captured by targeted chart review: type of pneumonitis (based on pneumonitis terms corresponding to the ICD code list), past radiotherapy with curative or palliative intent, timing of radiotherapy, duration, number of fractions, and total tumor dose of radiotherapy. For patients who were determined to have been diagnosed with treatment-associated pneumonitis, the use of corticosteroids for management of symptoms was captured.

#### RCT Cohort

Data were pooled from 14 randomized clinical trials that compared efficacy and safety outcomes for treatment with ICI (± chemotherapy) to chemotherapy alone in patients with aNSCLC. These 14 randomized clinical trials were identified from Drugs@FDA. The indications supported by these 14 trials were approved before December 31, 2020 (Supplementary Table S3). Subject-level data on demographics, disease stages, histology, smoking status etc., were obtained from FDA's internal electronic document room database. The terms for pneumonitis were identified using the Medical Dictionary for Regulatory Activities preferred terms: “pneumonitis,” “acute interstitial pneumonitis,” “pneumonitis chemical,” “radiation pneumonitis,” and “interstitial lung disease.” Pneumonitis cases diagnosed after the index treatment start date and within 30 days of the last administration of the index treatment were considered as treatment-associated pneumonitis, consistent with the methodology used with the real-world TAP cases. Sensitivity analyses were also performed to assess longer windows for defining TAP, and to assess high-grade TAP as defined by cases with grades ≥2 and grades ≥3 pneumonitis based on the Common Terminology Criteria for Adverse Events. Pneumonitis cases as defined above, diagnosed prior to index treatment start date, were considered as prior medical history of pneumonitis.

### Statistical Analyses

Descriptive statistics were used to summarize demographic and clinical characteristics of patients, stratified by treatment group and across all RWD patients and RCT patients. Radiotherapy characteristics among patients with suspected pneumonitis identified by structured data were summarized for the RWD patients. Incidence proportions were calculated as the number of TAP cases divided by the total number of patients in each treatment group. Stratified analyses were conducted to provide results for patients with and without a past medical history (PMH) of pneumonitis. These primary analyses were conducted for both the RCT patient cohort and the RWD patient cohort. Confidence intervals for incidence proportions were calculated on the basis of the Wilson method for binomial probabilities. All analyses were performed in R programming language, version 3.3.2 (R Foundation for Statistical Computing).

### Research Ethics

This study was exempt from Institutional Review Board approval because the study was retrospective, noninterventional, and used deidentified data.

### Data Availability Statement

The dataset generated and analyzed for the current study are proprietary and therefore, not publicly available.

## Results

The RWD cohort consisted of 1,723 patients diagnosed with aNSCLC, of whom 718 were in the ICI group and 1,005 were in the chemotherapy group. The index treatment initiation dates for the ICI treatment group occurred between 2015 and 2020, and the index treatment initiation dates for the chemotherapy treatment group occurred between 2010 and 2020. The characteristics of patients in these two treatment arms were comparable for sex, race, age at advanced and/or metastatic diagnosis, age at treatment index date, histology subtypes, and smoking status (although there is substantial data missing for smoking status; Table 1). A similar proportion of patients in each treatment group had metastatic disease after progression from localized disease (16%). ICI-treated patients were more likely to have a history of chronic lung disease (49% vs. 41%). ICI-treated patients had a shorter median follow-up time (8.3 vs. 11.4 months), while they were less likely to have died by the study cut-off date (59% vs. 77%). A larger proportion of patients with a PMH of pneumonitis also had a history of chronic lung disease compared with patients without a PMH of pneumonitis (74% vs. 44%). No patients were excluded because of missing covariates; any missingness was noted as a separate category in Table 1.

**TABLE 1:** Demographic and clinical characteristics of patients with advanced and/or metastatic NSCLC in the RWD cohort

	Immune checkpoint inhibitors (*N* = 718)	Chemotherapies (*N* = 1,005)	All RWD (*N* = 1,723)
Age at adv/met diagnosis, Median (Q1, Q3)	66 (59, 73)	66 (58, 74)	66 (58, 74)
Age at treatment index date, Median (Q1, Q3)	67 (59, 74)	66 (58, 74)	66 (59, 74)
Age categories at treatment index date, Count (%)			
≤49	29 (4%)	49 (5%)	78 (5%)
50–64	258 (36%)	379 (38%)	637 (37%)
65–74	218 (30%)	287 (29%)	505 (29%)
≥75	213 (30%)	290 (29%)	503 (29%)
Sex, Count (%)			
Female	332 (46%)	485 (48%)	817 (47%)
Male	386 (54%)	520 (52%)	906 (53%)
Race, Count (%)			
White	646 (90%)	858 (85%)	1,504 (87%)
Black or African American	56 (8%)	116 (12%)	172 (10%)
Asian	7 (1%)	8 (1%)	15 (1%)
Other	3 (0%)	17 (2%)	20 (1%)
Unknown/Not provided	6 (1%)	6 (1%)	12 (1%)
Stage at diagnosis, Count (%)			
I–II	7 (1%)	3 (0%)	10 (1%)
IIIA	104 (14%)	314 (31%)	418 (24%)
IIIB	64 (9%)	148 (15%)	212 (12%)
IIIC	8 (1%)	0 (0%)	8 (0%)
IV	535 (75%)	540 (54%)	1,075 (62%)
Metastatic diagnosis, Count (*N*%)			
*De novo*	535 (75%)	540 (54%)	1,075 (62%)
Progressed	113 (16%)	159 (16%)	272 (16%)
No metastasis	70 (10%)	306 (30%)	376 (22%)
Histology, Count (%)			
Adenocarcinoma	440 (61%)	587 (58%)	1,027 (60%)
Squamous cell carcinoma	179 (25%)	291 (29%)	470 (27%)
Large cell carcinoma	4 (1%)	13 (1%)	17 (1%)
Other	95 (13%)	114 (11%)	209 (12%)
Smoking status, Count (%)			
Never	27 (4%)	20 (2%)	47 (3%)
Former	208 (29%)	98 (10%)	306 (18%)
Current	93 (13%)	65 (6%)	158 (9%)
Unknown/Not provided	390 (54%)	822 (82%)	1,212 (70%)
Past history of pneumonitis, Count (%)	32 (4%)	11 (1%)	43 (2%)
Past history of any chronic lung disease, Count (%)	354 (49%)	412 (41%)	766 (44%)

The RCT cohort consisted of 10,953 patients diagnosed with aNSCLC: 6,408 in the ICI group and 4,545 in the chemotherapy group. The index treatment initiation dates were between 2013 and 2019. The characteristics of patients in these two treatment arms were comparable for sex, race, age at treatment index date, histology subtypes, smoking status, and cancer stage at diagnosis (Table 2).

**TABLE 2:** Demographic and clinical characteristics of patients with advanced and/or metastatic NSCLC in the RCT cohort

	Immune checkpoint inhibitors (*N* = 6,408)	Chemotherapies (*N* = 4,545)	All RCT (*N* = 10,953)
Age at treatment index date, Median (Q1, Q3)	64 (57,69)	64 (57,70)	64 (57,69)
Age categories at treatment index date, Count (%)			
≤49	535 (8%)	348 (8%)	883 (8%)
50–65	2,922 (46%)	2,068 (46%)	4,990 (46%)
65–74	2,311 (36%)	1,685 (36%)	3,996 (36%)
≥75	640 (10%)	444 (10%)	1,084 (10%)
Sex, Count (%)			
Female	2,249 (35%)	1,586 (35%)	3,835 (35%)
Male	4,159 (65%)	2,959 (65%)	7,118 (65%)
Race, Count (%)			
White	5,043 (79%)	3,582 (79%)	8,625 (79%)
Black or African American	122 (2%)	92 (2%)	214 (2%)
Asian	1,028 (16%)	728 (16%)	1,756 (16%)
Other	119 (2%)	78 (2%)	197 (2%)
Unknown/Not provided	96 (1.5%)	65 (1.4%)	161 (1.5%)
Stage at diagnosis, Count (%)			
I	129 (2%)	111 (2%)	240 (2%)
II	136 (2%)	97 (2%)	233 (2%)
III	504 (8%)	412 (9%)	916 (8%)
IV	5,446 (85%)	3,821 (84%)	9,267 (84%)
Recurrent	193 (3%)	104 (2%)	297 (3%)
Histology, Count (%)			
Adenocarcinoma	4,031 (63%)	2,552 (56%)	6,583 (60%)
Squamous cell carcinoma	1,332 (21%)	1,077 (24%)	2,409 (22%)
Large cell carcinoma	70 (1.1%)	35 (0.8%)	105 (1%)
Other	939 (15%)	854 (19%)	1,793 (16%)
Unknown	36 (0.6%)	27 (0.6%)	63 (1%)
Smoking status, Count (%)			
Never	1,012 (16%)	707 (16%)	1,719 (16%)
Former	2,390 (37%)	1,808 (40%)	4,198 (38%)
Current	788 (12%)	626 (14%)	1,414 (13%)
Current/Former	2,195 (34%)	1,388 (31%)	3,583 (33%)
Unknown/ Not provided	23 (0.4%)	16 (0.4%)	39 (0.4%)
Past medical history of pneumonitis, Count (%)	39 (1%)	27 (1%)	66 (1%)

**FIGURE 1:**
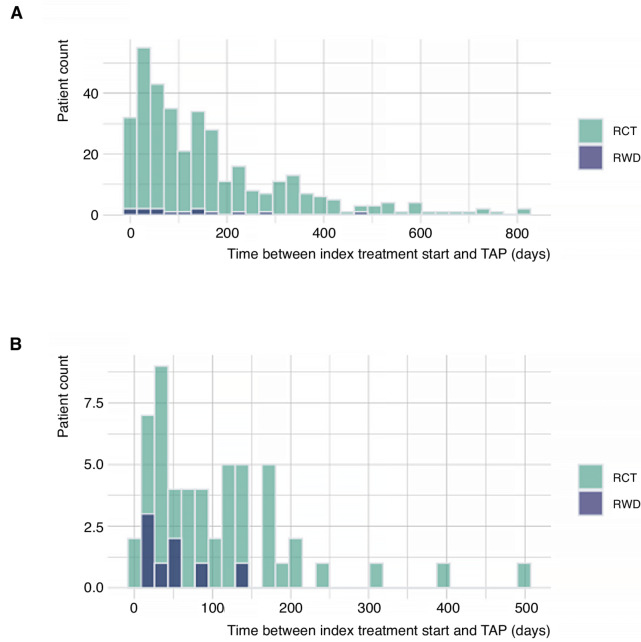
Distribution of time from index treatment start to TAP^a^ diagnosis date in ICI- (**A**), and chemotherapy-treated (**B**) patients. ^a^Defined as pneumonitis cases diagnosed after the index treatment start date and within 30 days of the last administration of the index treatment.

Among RWD patients, 14 of the 718 ICI-treated patients (1.9%; 95% CI, 1.2–3.2) and 8 of the 1,005 chemotherapy-treated patients (0.8%; 95% CI, 0.4–1.6) were diagnosed with TAP (Table 3). A large proportion of TAP cases were managed with corticosteroids (12/14 ICI-treated patients, 7/8 chemotherapy-treated patients). Among the 22 RWD patients with TAP, the median number of days from the index treatment start date to TAP was 60 days [98 days in the ICI treatment group and 47 days in the chemotherapy treatment group (Fig. 1)]. Among both ICI and chemotherapy groups a higher incidence of TAP was observed among patients with a PMH of pneumonitis as compared with those without a PMH of pneumonitis (Table 3). Incidence estimates did not vary greatly when windows for defining TAP were lengthened from 30 to 60 or 90 days (Table 3; Supplementary Table S4). In the RWD analysis, the median time between PMH of pneumonitis and the index treatment start date was 359 days for the ICI treatment group and 351 days for the chemotherapy treatment group. In the sensitivity analysis restricting the chemotherapy treatment group duration to 2015–2020 to approximate the same duration observed in ICI-treated patients, TAP rate was 0.4% compared with 0.8% from the full study period of 2010–2020.

**TABLE 3:** Incidence of TAP^a^ in the RCT cohort and the RWD cohort

	RCT immune checkpoint inhibitors (*N* = 6,408)	RCT chemotherapies (*N* = 4,545)	RWD immune checkpoint inhibitors (*N* = 718)	RWD chemotherapies (*N* = 1,005)
Patients with past medical history of pneumonitis (*N* = 66 for RCT; *N* = 43 for RWD)	6/39 (15.4%; 7.3–29.7)	2/27 (7.4%; 2.1–23.4)	4/32 (12.5%; 5.0–28.0)	1/11 (9.1%; 0.5–37.7)
Patients without past medical history of pneumonitis (*N* = 10,887 for RCT; *N* = 1,680 for RWD)	351/6,369 (5.5%; 5.0–6.1)	52/4,518 (1.2%; 0.9–1.5)	10/686 (1.5%; 0.8–2.7)	7/994 (0.7%; 0.3–1.4)
All patients (*N* = 10,953 for RCTs; *N* = 1,723 for RWD)	357/6,408 (5.6%; 5.0–6.2)	54/4,545 (1.2%; 0.9–1.5)	14/718 (1.9%; 1.2–3.2)	8/1,005 (0.8%; 0.4–1.6)

^a^Defined as pneumonitis cases diagnosed after the index treatment start date and within 30 days of the last administration of the index treatment.

Among RCT patients, 357 of 6,408 ICI-treated patients (5.6%; 95% CI, 5.0–6.2) and 54 of 4,545 chemotherapy-treated patients (1.2%; 95% CI, 0.9–1.5) were diagnosed with TAP (Table 3). The median time from the index treatment date to TAP diagnosis date was 108 days in 411 RCT patients with TAP, with a median time of 115 days in the ICI group and 78 days in the chemotherapy group (Fig. 1). A large proportion of TAP cases in the RCT cohort were managed with corticosteroids (281/357 ICI-treated patients, 39/54 chemotherapy-treated patients). Similar to the RWD cohort, a higher incidence of TAP was observed among patients with a PMH of pneumonitis compared with those without PMH of pneumonitis (Table 3). Incidence estimates did not vary significantly for either treatment arm when windows for defining TAP were lengthened from 30 to 90 days (Table 3; Supplementary Table S4). Compared with overall pneumonitis rates, incidence rates of TAP were lower when cases were restricted to TAP grades ≥2 (4.3% and 1.0% for ICI and chemotherapy groups, respectively) and TAP grades ≥3 (2.0% and 0.6% for ICI and chemotherapy groups, respectively; Supplementary Table S5). Among RCT patients with TAP grades ≥3, the median times from the index treatment date to TAP diagnosis date were 67 days in the ICI group and 76 days in the chemotherapy group.

Within the RWD cohort, 100% of the TAP-diagnosed patients in both treatment groups had received thoracic radiotherapy prior to TAP, and 55% of the TAP-diagnosed patients had received thoracic radiotherapy prior to index treatment In contrast, 25% of the subset (*n* = 821) of RWD patients with available radiotherapy status had received thoracic radiotherapy prior to index treatment. This subset of 821 patients with available radiotherapy status was not substantially different from the general RWD cohort (i.e., age at diagnosis, sex, race, and follow-up time) except for metastatic status (Supplementary Table S6). A majority of patients in both treatment groups were diagnosed with TAP that was clinically attributed to radiation pneumonitis within the electronic medical record (64% in the ICI treatment group and 63% in the chemotherapy group; Table 4). For patients with TAP attributed to radiation pneumonitis, the median time from prior radiotherapy to TAP was 130 days (Q1–Q3: 129–132). No patient deaths were attributed to pneumonitis in either RWD or RCT cohorts.

**TABLE 4:** Characterization of patients with TAP^a^ in the RWD cohort

	Immune checkpoint inhibitors (*N* = 14)	Chemotherapies (*N* = 8)	All RWD (*N* = 22)
TAP attributed to radiation pneumonitis	9 (64%)	5 (63%)	14 (64%)
Received radiotherapy^b^ prior to index treatment	9 (64%)	3 (38%)	12 (55%)
Received radiotherapy^b^ prior to TAP	14 (100%)	8 (100%)	22 (100%)
Received radiaotherapy with curative intent prior to TAP	8 (57%)	5 (63%)	13 (59%)
History of radiation pneumonitis prior to TAP	3 (21%)	0 (0%)	3 (14%)
Past medical history of pneumonitis prior to index treatment	4 (29%)	1 (13%)	5 (23%)

^a^Defined as pneumonitis cases diagnosed after the index treatment start date and within 30 days of the last administration of the index treatment.

^b^Received with either curative or palliative intent to the primary site (lung/thorax).

## Discussion

In this analysis of TAP incidence among patients with NSCLC treated with ICI or chemotherapy, we observed a higher rate of TAP diagnosis in the RCT cohort as compared with the RWD cohort. This differs from previous reports where RWD analyses reported higher incidence compared with RCT (11, 12, 19). However, estimates of higher severity (≥ grade 3) TAP in the RCT cohort were similar to that of the overall RWD cohort, suggesting that asymptomatic and minimally symptomatic TAP cases may be underestimated or less well captured in our RWD cohort. In both RCT and RWD cohorts, TAP incidence was numerically higher among the ICI-treated patients compared with the chemotherapy-treated patients, although the difference was not significant in the RWD cohort. Higher rates of documented TAP in patients with a prior medical history of pneumonitis (although sample size is limited for the RWD analysis) were observed regardless of index therapy type or patient cohort. No patient deaths were attributed to pneumonitis in either RWD or RCT cohorts.

In the RWD cohort, all patients with TAP had received thoracic radiation prior to TAP, and 55% of patients with TAP received thoracic radiation prior to index therapy. Radiation treatment status was not available for the entire general RWD cohort; however, it was available for a subset of 821 patients. In this subset of the general RWD cohort (including both patients with and without TAP), a lower proportion of patients (25%) received thoracic radiotherapy prior to index treatment compared with TAP-diagnosed patients. This suggests a potential connection between prior thoracic radiation and TAP. No substantial differences were observed between the subsets where radiotherapy status was available or not, with the exception of a higher proportion of patients with metastatic disease (at index treatment) in the subset with radiotherapy status available. Given that patients with metastatic disease are less likely to receive thoracic irradiation, especially with curative intent, use of this subset of patients as a representative sample may lead to an underestimation of the past receipt of thoracic radiotherapy in the RWD cohort. Because of questions around generalizability, potential confounding, and other differences between patient groups with or without tumor registry data, additional studies are needed to further assess the association between TAP and prior thoracic radiotherapy.

The use of ICIs for the treatment of aNSCLC has increased, alongside the need for better data describing ICI-mediated pneumonitis, including the incidence, time to development, severity, and potential predisposing patient factors. Although clinical trials tend to report relatively lower pneumonitis rates among ICI-treated patients (3%–5% of patients) compared with previously reported RWD studies (15–18), the latter have reported much wider ranges of pneumonitis incidence (4%–19%), making it difficult to generalize risk in the real-world setting (11, 12, 19, 24). In addition to previously mentioned factors likely contributing to this variability between and within RCT and RWD (e.g., varying estimates with respect to study methodology, data availability, histology, specific ICI agents assessed, monotherapy vs. multiagent or multimodality regimens, prior treatment history, increasing clinician awareness over time regarding TAP diagnosis and associated risk factors, etc.; refs. 10–12, 16, 19, 20), another important factor is the lack of a single consensus–based approach to identifying adverse drug event occurrences and attribution in the RWD setting. Pneumonitis is a diagnosis of exclusion, for example, ruling out infection (based on no use of antibiotics or negative cultures), disease progression, or other etiologies, in the presence of symptoms such as dyspnea, cough, or shortness of breath. Given the lack of a definitive diagnostic test, variability across studies in the operational definition of TAP and the level of clinical detail available in the source data may result in differing classifications of patients and either underestimation or overestimation of TAP incidence. While the true incidence cannot be definitively stated, the consistent use of a consensus definition for TAP would improve the ability to compare incidences across studies. An example of potential confounding diagnoses is noted in a report that described a 9.5% incidence (30/315 patients) of ICI-related pneumonitis, but with 70% of the pneumonitis patients having received empiric antimicrobial therapy (25). This underscores the challenge of distinguishing between infectious and noninfectious causes of pulmonary inflammation.

Another challenge in the assessment of treatment-related pneumonitis is how to assess patients labeled or diagnosed as having radiation pneumonitis. Some studies evaluating ICI therapies have explicitly excluded patients labeled as having radiation pneumonitis from being designated as TAP (25, 26). In this study, patients identified as having radiation pneumonitis were designated as TAP in both cohorts because: (i) radiation pneumonitis is largely a clinical diagnosis; and (ii) radiation recall might be triggered by the anticancer treatment. While radiation recall is mainly observed with chemotherapeutic agents with median time of onset of 95 days after the end of radiotherapy (27), other agents including ICIs have also been reported as associated with radiation recall, with onset as long as 2 years after radiotherapy (23, 28–31). It should be noted that were one to exclude patients diagnosed as having radiation pneumonitis within the window of observation in our study, the rates of TAP would have been 0.7% and 0.3% for ICI-treated and chemotherapy-treated patients, respectively, within the RWD cohort. Deciding how to attribute TAP in these patients labeled as having radiation pneumonitis is difficult: the underlying cause of the pneumonitis is often not definitively proven, and therapies can have overlapping or synergistic toxicities. Given this, the need for a consensus definition of TAP for RWD becomes even more critical.

Differences in patient identification periods may also contribute to differences in observed TAP incidence between studies (whether RWD or RCT). Earlier published studies assessing patients treated prior to the approval of PD-L1 inhibitors report rates for a different distribution of ICIs compared with our more recent RWD study of patients who received one of four available ICIs as recently as September 2020. Finally, differences between earlier and more recent use of ICIs for treatment of NSCLC, including use of supportive care and patient selection for treatment may have shifted over time as physicians gain experience using ICIs with their patients with aNSCLC, resulting in lower TAP seen in this more recent study.

The results of this study should be interpreted within the context of inherent limitations. To our knowledge, our study cohort is the largest U.S. real-world population studied to date; however, the sample size is still relatively small compared with the pooled RCT analysis. Because of these limitations of the data, descriptive analyses are unadjusted for potentially confounding patient characteristics such as history of chronic lung disease and line of therapy of index treatment, which does not allow any inference of causality in this study (32). Although TAP incidence was higher among patients with a history of pneumonitis compared with those without, it is possible that the risk factor is not a history of pneumonitis, but the common underlying predisposing factor of chronic lung disease (a correlate of PMH of pneumonitis in the RWD cohort) or accumulated insults to the lungs including the effects of smoking, infection, drug therapy, or radiation. Another potential contributing factor to the higher TAP incidence among patients with PMH of pneumonitis may be a higher likelihood of clinicians to diagnose pneumonitis in patients with a history of lung disease (including PMH of pneumonitis), that is, diagnostic bias. Several factors may contribute to the differences in TAP seen between RCT and RWD. Close clinical monitoring and observation of patients in RCTs (i.e., well-defined, prespecified imaging and follow-up assessment schedule), could potentially lead to better detection of TAP in the RCT setting. On the other hand, clinical trial participants are subject to more stringent eligibility criteria, resulting in a selection of patients with fewer comorbidities, potentially leading to a group of patients with lower predisposition to TAP compared with RWD. In addition, our study approach of using ICD codes to initially identify TAP naturally selected for patients with more prominent clinical manifestations of TAP. This may in part explain the lower incidence of overall (i.e., all-grade) TAP observed in this RWD cohort compared with other studies in the real-world setting for which the review of charts, radiology reports and/or imaging were conducted for the entire cohort (25). Among previous RWD studies that reported both all-grade and high-grade cases, high-grade (grades ≥3) pneumonitis rates were substantially lower (3.4%–5.6%) compared with all-grade pneumonitis rates (13.2%–19%; refs. 11, 12, 25, 33). When evaluating patients with TAP of grade ≥3, the RCT estimates in this study showed lower TAP rates of 2.0% and 0.6% for ICI-treated and chemotherapy-treated patients, respectively, similar to the overall RWD estimates in this study of 1.9% and 0.8%, respectively. Consistent with these findings, time to TAP among all-grade patients in the RCT cohort was longer compared with that in the RWD cohort, whereas restriction to grade 3+ RCT patients resulted in shorter and more comparable time to TAP as in the RWD setting. This further suggests that TAP cases in this cohort of RWD patients are likely higher grade compared with the general RCT cohort, with quicker manifestation of TAP compared with the asymptomatic and minimally symptomatic cases more likely to be identified through regularly scheduled imaging in the RCT cohort. Another consideration for the interpretation of the findings of this study is that the ICI and chemotherapy treatment groups differ on clinically important characteristics, in that the ICI treatment group could have received prior chemotherapy for advanced and/or metastatic disease, whereas the chemotherapy treatment group represents the first chemotherapy exposure for advanced and/or metastatic disease. Finally, although a diverse population, patients in the RWD analysis are from the Midwestern United States whereas patients in the RCT analysis were from a broader U.S. population and included international, multicenter trials as well. Therefore, there may be limitations in comparability between RWD and RCT patients, and additionally between RWD and the broader U.S. population, respectively.

As immunotherapies take on an increasingly prominent role in the treatment landscape for a wide range of cancers, there is an important need to better understand the incidence of and risk factors for TAP in real-world practice. In 2020, 4,720 immuno-oncology drugs were in the global development pipeline, a 22% expansion from 2019, despite the impact of the COVID pandemic (34). The proliferation of research and clinical trials to evaluate efficacy and safety of new and existing ICIs offers new hope for many patients with cancer; however, it also necessitates the need to fully understand safety considerations, including TAP and other adverse drug events. Real-world data can serve as an additional source of valuable information to complement clinical trial data to better understand potential adverse events and to improve the care of patients with cancer. However, to best use RWD for understanding outcomes in routine clinical care, there are methodologic challenges that need to be addressed. Variability in findings across RWD studies for TAP highlights the need to improve source data quality and consensus definitions for clinical diagnoses like pneumonitis to increase confidence in the results from observational studies.

## Supplementary Material

Supplementary Tables 1-6 Supplementary Table 1. Pneumonitis defined using A) ICD-9 and B) ICD-10 codes that included ‘pneumonitis’ or associated concepts in the Unified Medical Language System, with Bidirectional General Equivalence Mappings used to identify equivalent terms between ICD-9 and ICD-10 in the Real World Data (RWD) cohort
Supplementary Table 2: ICD codes used to identify history of chronic lung condition in the Real World Data (RWD) cohort
Supplementary Table 3. Selection of clinical trial (RCT) population
Supplementary Table 4. Incidence of Treatment-Associated Pneumonitis in the Randomized Clinical Trial (RCT) Cohort and the Real World Data (RWD) Cohort based on sensitivity analyses
Supplementary Table 5. Treatment-associated pneumonitis counts by grade in the Randomized Clinical Trial (RCT) cohort
Supplementary Table 6. Demographic and Clinical Characteristics of Patients With and Without Radiation Therapy (RT) status available in the Real World Data (RWD) Cohort
