# Supplementary material for: Evaluating Pneumonitis Incidence in Patients with Non–small Cell Lung Cancer Treated with Immunotherapy and/or Chemotherapy Using Real-world and Clinical Trial Data
Source: Cancer Res Commun. 2023 Feb 14;3(2):258–66. doi: 10.1158/2767-9764.CRC-22-0370 (PMC9973394; doi:10.1158/2767-9764.CRC-22-0370)
Supplement: Supplementary Tables 1-6 — Supplementary Table 1. Pneumonitis defined using A) ICD-9 and B) ICD-10 codes that included ‘pneumonitis’ or associated concepts in the Unified Medical Language System, with Bidirectional General Equivalence Mappings used to identify equivalent terms between ICD-9 and ICD-10 in the Real World Data (RWD) cohort Supplementary Table 2: ICD codes used to identify history of chronic lung condition in the Real World Data (RWD) cohort Supplementary Table 3. Selection of clinical trial (RCT) population Supplementary Table 4. Incidence of Treatment-Associated Pneumonitis in the Randomized Clinical Trial (RCT) Cohort and the Real World Data (RWD) Cohort based on sensitivity analyses Supplementary Table 5. Treatment-associated pneumonitis counts by grade in the Randomized Clinical Trial (RCT) cohort Supplementary Table 6. Demographic and Clinical Characteristics of Patients With and Without Radiation Therapy (RT) status available in the Real World Data (RWD) Cohort [file crc-22-0370-s01.pdf]

Supplementary Table 1. Pneumonitis defined using A) ICD-9 and B) ICD-10 codes that included 'pneumonitis' or associated concepts in the Unified Medical Language System, with Bidirectional General Equivalence Mappings used to identify equivalent terms between ICD-9 and ICD-10 in the Real World Data (RWD) cohort

A) ICD-9 codes

Term	Description	Code System
052.1	Varicella (hemorrhagic) pneumonitis	ICD-9-CM
130.4	Pneumonitis due to toxoplasmosis	ICD-9-CM
495.7	"Ventilation" pneumonitis	ICD-9-CM
507.0	Pneumonitis due to inhalation of food or vomitus	ICD-9-CM
507.1	Pneumonitis due to inhalation of oils and essences	ICD-9-CM
507.8	Pneumonitis due to other solids and liquids	ICD-9-CM
516.32	Idiopathic non-specific interstitial pneumonitis	ICD-9-CM
516.33	Acute interstitial pneumonitis	ICD-9-CM
484.1	Pneumonia in cytomegalic inclusion disease	ICD-9-CM
495.0	Farmers' lung	ICD-9-CM
495.1	Bagassosis	ICD-9-CM
495.2	Bird-fancier's lung	ICD-9-CM
495.3	Suberosis	ICD-9-CM
495.4	Malt workers' lung	ICD-9-CM
495.5	Mushroom workers' lung	ICD-9-CM
495.6	Maple bark-strippers' lung	ICD-9-CM
495.8	Other specified allergic alveolitis and pneumonitis	ICD-9-CM
495.9	Unspecified allergic alveolitis and pneumonitis	ICD-9-CM
516.35	Idiopathic lymphoid interstitial pneumonia	ICD-9-CM
997.31	Ventilator associated pneumonia	ICD-9-CM
770.12	Meconium aspiration with respiratory symptoms	ICD-9-CM

B) ICD-10 codes

Term	Code	Code System

Acute interstitial pneumonitis	J84.114	ICD-10-CM
Air Conditioner and humidifier lung	J67.7	ICD-10-CM
Allergis Alveolitis	J67	ICD-10-CM
Alveolitis due to Aspergillus clavatus	J67.4	ICD-10-CM
Alveolitis due to Cryptostroma corticale	J67.6	ICD-10-CM
Aspiration pneumonia	J69.0	ICD-10-CM
Aspiration pneumonitis due to anesthesia during delivery	O74.0	ICD-10-CM
Aspiration pneumonitis due to anesthesia during pregnancy	O29.01	ICD-10-CM
Aspiration pneumonitis due to anesthesia during pregnancy [multiple terms[	O29.011	ICD-10-CM
Aspiration pneumonitis due to anesthesia during pregnancy [multiple terms[	O29.012	ICD-10-CM
Aspiration pneumonitis due to anesthesia during pregnancy [multiple terms[	O29.013	ICD-10-CM
Aspiration pneumonitis due to anesthesia during pregnancy [multiple terms[	O29.019	ICD-10-CM
Aspiration pneumonitis due to anesthesia during the puerperium	O89.01	ICD-10-CM
Bagasse disease	J67.1	ICD-10-CM
Bagasse pneumonitis	J67.1	ICD-10-CM
Bagassosis	J67.1	ICD-10-CM
Bird fancier's lung	J67.2	ICD-10-CM
Budgerigar fancier's disease or lung	J67.2	ICD-10-CM
Cheese-washer's lung	J67.8	ICD-10-CM
Chemical pneumonitis due to anesthesia	J95.4	ICD-10-CM
Coffee-worker's lung	J67.8	ICD-10-CM
Corkhandler's disease or lung	J67.3	ICD-10-CM
Corkworker's disease or lung	J67.3	ICD-10-CM
Cryptostromosis	J67.6	ICD-10-CM
Cytomegaloviral pneumonitis	B25.0	ICD-10-CM

Exogenous lipoid pneumonia	J69.1	ICD-10-CM
Farmer's Lung	J67.0	ICD-10-CM
Fish-meal worker's lung	J67.8	ICD-10-CM
Furrier's lung	J67.8	ICD-10-CM
Hamman-Rich syndrome	J84.114	ICD-10-CM
Harvester's Lung	J67.0	ICD-10-CM
Haymaker's Lung	J67.0	ICD-10-CM
Hypersensitivity pneumonitis due to organic dust	J67	ICD-10-CM
Hypersensitivity pneumonitis due to other organic dusts	J67.8	ICD-10-CM
Hypersensitivity pneumonitis due to unspecified organic dust	J67.9	ICD-10-CM
Idiopathic non-specific interstitial pneumonitis	J84.113	ICD-10-CM
Inhalation of stomach contents or secretions NOS due to anesthesia during pregnancy	O29.01	ICD-10-CM
Interstitial pneumonitis	J84.89	ICD-10-CM
Lipid pneumonia	J69.1	ICD-10-CM
Lymphoid interstitial pneumonia	J84.2	ICD-10-CM
Lymphoid Interstitial pneumonitis	J84.2	ICD-10-CM
Maltworker's lung	J67.4	ICD-10-CM
Maple-bark-stripper's lung	J67.6	ICD-10-CM
Meconium aspiration pneumonia	P24.01	ICD-10-CM
Meconium aspiration pneumonitis	P24.01	ICD-10-CM
Meconium aspiration syndrome	P24.01	ICD-10-CM
Meconium aspiration with respiratory symptoms	P24.01	ICD-10-CM
Mendelson's syndrome	J95.4	ICD-10-CM
Mendelson's syndrome due to anesthesia during delivery	O74.0	ICD-10-CM
Mendelson's syndrome due to anesthesia during pregnancy	O29.01	ICD-10-CM
Mendelson's syndrome due to anesthesia during the puerperium	O89.01	ICD-10-CM
Moldy hay disease	J67.0	ICD-10-CM

Mushroom-worker's lung	J67.5	ICD-10-CM
Pigeon fancier's disease or lung	J67.2	ICD-10-CM
Pneumonitis due to inhalation of oils and essences	J69.1	ICD-10-CM
Pneumonitis due to inhalation of other solids and liquids	J69.8	ICD-10-CM
Pneumonitis due to solids and liquids	J69	ICD-10-CM
Postprocedural aspiration pneumonia	J95.4	ICD-10-CM
Radiation pneumonitis	J70.0	ICD-10-CM
Sequoiosis	J67.8	ICD-10-CM
Suberosis	J67.3	ICD-10-CM
Ventilator associated pneumonia	J95.851	ICD-10-CM
Ventilator associated pneumonitis	J95.851	ICD-10-CM

Supplementary Table 2: ICD codes used to identify history of chronic lung condition in the Real World Data (RWD) cohort

ICD 10	Description
J44.9	Chronic obstructive pulmonary disease, unspecified
J44.1	Chronic obstructive pulmonary disease w (acute) exacerbation
J40	Bronchitis, not specified as acute or chronic
J45.909	Unspecified asthma, uncomplicated
I26.99	Other pulmonary embolism without acute cor pulmonale
J43.9	Emphysema, unspecified
G47.33	Obstructive sleep apnea (adult) (pediatric)
J43.2	Centrilobular emphysema
D38.1	Neoplasm of uncertain behavior of trachea, bronchus and lung
G47.30	Sleep apnea, unspecified
J45.20	Mild intermittent asthma, uncomplicated
J43.8	Other emphysema
I27.20	Pulmonary hypertension, unspecified
J43.1	Panlobular emphysema
J42	Unspecified chronic bronchitis
J44.0	Chronic obstructive pulmon disease w acute lower resp infct
C33	Malignant neoplasm of trachea
J45.21	Mild intermittent asthma with (acute) exacerbation
J45.901	Unspecified asthma with (acute) exacerbation
J45.40	Moderate persistent asthma, uncomplicated
J45.30	Mild persistent asthma, uncomplicated
J45.41	Moderate persistent asthma with (acute) exacerbation
J41.0	Simple chronic bronchitis
I27.2	Other secondary pulmonary hypertension
G47.34	Idio sleep related nonobstructive alveolar hypoventilation
J92.9	Pleural plaque without asbestos
I27.82	Chronic pulmonary embolism
J33.9	Nasal polyp, unspecified

J41.1	Mucopurulent chronic bronchitis
J45.31	Mild persistent asthma with (acute) exacerbation
I26.90	Septic pulmonary embolism without acute cor pulmonale
I27.0	Primary pulmonary hypertension
I27.81	Cor pulmonale (chronic)
J47.9	Bronchiectasis, uncomplicated
J41.8	Mixed simple and mucopurulent chronic bronchitis
J45.991	Cough variant asthma
C32.0	Malignant neoplasm of glottis
D38.6	Neoplasm of uncertain behavior of respiratory organ, unsp
D86.0	Sarcoidosis of lung
D86.9	Sarcoidosis, unspecified
G47.36	Sleep related hypoventilation in conditions classd elswhr
I27.21	Secondary pulmonary arterial hypertension
J33.0	Polyp of nasal cavity
J45.50	Severe persistent asthma, uncomplicated
J45.51	Severe persistent asthma with (acute) exacerbation
Q33.8	Other congenital malformations of lung
A15.0	Tuberculosis of lung
A15.7	Primary respiratory tuberculosis
A15.9	Respiratory tuberculosis unspecified
C32.9	Malignant neoplasm of larynx, unspecified
G47.31	Primary central sleep apnea
G47.39	Other sleep apnea
I26.01	Septic pulmonary embolism with acute cor pulmonale
I26.02	Saddle embolus of pulmonary artery with acute cor pulmonale
I26.09	Other pulmonary embolism with acute cor pulmonale
I26.92	Saddle embolus of pulmonary artery w/o acute cor pulmonale
I26.93	Single subsegmental pulmonary embolism without acute cor pulmonale
I26.94	Multiple subsegmental pulmonary emboli without acute cor pulmonale
I27.23	Pulmonary hypertension due to lung diseases and hypoxia

I27.9	Pulmonary heart disease, unspecified
J43.0	Unilateral pulmonary emphysema [MacLeod's syndrome]
J45.902	Unspecified asthma with status asthmaticus
J45.990	Exercise induced bronchospasm
J47.1	Bronchiectasis with (acute) exacerbation
J61	Pneumoconiosis due to asbestos and other mineral fibers
J62.8	Pneumoconiosis due to other dust containing silica
J82	Pulmonary eosinophilia, not elsewhere classified
J92.0	Pleural plaque with presence of asbestos
Q33.6	Congenital hypoplasia and dysplasia of lung
Q33.9	Congenital malformation of lung, unspecified

Supplementary Table 3. Selection of clinical trial (RCT) population

Trial name	Experimental Arm	Control Arm
KEYNOTE-189 (NCT02578680)	Pembrolizumab + carboplatin or cisplatin and pemetrexed	Saline placebo + carboplatin or cisplatin and pemetrexed
KEYNOTE-407 (NCT02775435)	Pembrolizumab + Carboplatin + Paclitaxel or Nab-Paclitaxel	Saline placebo + Carboplatin + Paclitaxel or Nab-Paclitaxel
KEYNOTE-042 (NCT02220894)	Pembrolizumab	Chemotherapy Standard of Care
KEYNOTE-024 (NCT02142738)	Pembrolizumab	Chemotherapy Standard of Care
KEYNOTE-010 (NCT01905657)	Pembrolizumab	Docetaxel
CHECKMATE-227 (NCT02477826)	Nivolumab or Nivolumab + Lpilimumab or Nivolumab + Platinum doublet chemotherapy	Platinum-Doublet Chemotherapy
CHECKMATE-9LA (NCT03215706)	Nivolumab + Lpilimumab + Platinum-Doublet Chemotherapy	Platinum-Doublet Chemotherapy
CHECKMATE-017 (NCT01642004)	Nivolumab	Docetaxel
CHECKMATE-057 (NCT01673867)	Nivolumab	Docetaxel
IMpower110 (NCT02409342)	Atezolizumab	Platinum-Based Chemotherapy
IMpower150 (NCT02366143)	Atezolizumab + Paclitaxel + Carboplatin or Atezolizumab + Bevacizumab + Paclitaxel + Carboplatin	Bevacizumab + Paclitaxel + Carboplatin
IMpower130 (NCT02367781)	Atezolizumab + Nab-Paclitaxel + Carboplatin	Nab-Paclitaxel + Carboplatin
OAK (NCT02008227)	Atezolizumab	Docetaxel
POPLAR (NCT01903993)	Atezolizumab	Docetaxel
*PACIFIC study (NCT02125461)	Durvalumab	Placebo

* The control arm of PACIFIC study for Durvalumab is only placebo, thus PACIFIC study for Durvalumab is excluded

Supplementary Table 4. Incidence of Treatment-Associated Pneumonitis in the Randomized Clinical Trial (RCT) Cohort and the Real World Data (RWD) Cohort based on sensitivity analyses[a]

	**RCT** immune checkpoint inhibitors (N=6408)	**RCT** chemotherapies (N=4545)	**RWD** immune checkpoint inhibitors (N=718)	**RWD** chemotherapies (N= 1005)
Patients with past medical history of pneumonitis (N=66 for RCT; N=43 for RWD)	6/39 (15.4%; 7.3-29.7%)	2/27 (7.4%; 2.1-23.4%)	5/32 (15.6%; 6.9-31.8%)	1/11 (9.1%; 0.5-37.7%)
Patients without past medical history of pneumonitis (N=10887 for RCT; N=1680 for RWD)	365/6369 (5.7%; 5.2-6.3%)	54/4518 (1.2%; 0.9-1.6%)	10/686 (1.5%; 0.8-2.7%)	8/994 (0.8%; 0.4-1.6%)
All patients (N=10953 for RCTs; N=1723 for RWD)	371/6408 (5.8%; 5.2-6.4%)	56/4545 (1.2%; 1.0-1.6%)	15/718 (2.1%; 1.3-3.4%)	9/1005 (0.9%; 0.5-1.7%)

[a]TAP defined as pneumonitis cases diagnosed after the index treatment start date and within 90 days of the last administration of the index treatment

Supplementary Table 5. Treatment-associated pneumonitis counts by grade in the Randomized Clinical Trial (RCT) cohort

	Immune checkpoint inhibitor treatment group	Chemotherapy treatment group
RCT Cohort<br>All Grade TAP	357/6408<br>(5.7%; 5.0-6.2%)	54/4545<br>(1.2 %; 0.9-1.5%)
RCT Cohort<br>Grade 2+ patients	278/6408<br>(4.3%, 3.9%-4.9%)	47/4545<br>(1.0%, 0.8%-1.4%)
RCT Cohort<br>Grade 3+ patients	125/6408<br>(2.0%, 1.6%-2.3%)	28/4545<br>(0.6%, 0.4-0.9%)

Supplementary Table 6. Demographic and Clinical Characteristics of Patients With and Without Radiation Therapy (RT) status available in the Real World Data (RWD) Cohort

	RT status available (N=821)	RT status not available (N=902)	All RWD (N=1723)
**Age at adv/met diagnosis, Median (Q1, Q3)**	66.0 (58.5, 73.5)	65.3 (58.0, 73.5)	66 (58, 74)
**Age at treatment index date, Median (Q1, Q3)**	66.7 (59.1, 74.2)	65.8 (58.4, 73.6)	66 (59, 74)
**Age categories at treatment index date, Count (%)**			
<=49	34 (4%)	44 (5%)	78 (5%)
50-64	285 (35%)	352 (39%)	637 (37%)
65-74	248 (30%)	257 (28%)	505 (29%)
>=75	254 (31%)	249 (28%)	503 (29%)
**Sex, Count (%)**			
Female	398 (48%)	419 (46%)	817 (47%)
Male	423 (52%)	483 (54%)	906 (53%)
**Race, Count (%)**			
White	728 (89%)	776 (86%)	1504 (87%)
Black or African American	71 (9%)	101 (11%)	172 (10%)
Asian	8 (1%)	7 (1%)	15 (1%)
Other	5 (1%)	15 (2%)	20 (1%)
Unknown/Not Provided	9 (1%)	3 (0%)	12 (1%)
**Stage at diagnosis, Count (%)**			
I-II	10 (1%)	0 (0%)	10 (1%)
IIIA	145 (18%)	273 (30%)	418 (24%)
IIIB	98 (12%)	114 (13%)	212 (12%)
IIIC	6 (1%)	2 (0%)	8 (0%)
IV	562 (68%)	513 (57%)	1075 (62%)
**Metastatic diagnosis, Count (N%)**			
De novo	562 (68%)	513 (57%)	1075 (62%)
Progressed	143 (17%)	129 (14%)	272 (16%)
No metastasis	116 (14%)	260 (29%)	376 (22%)
**Histology, Count (%)**			
Adenocarcinoma	498 (61%)	529 (59%)	1027 (60%)

Squamous cell carcinoma	214 (26%)	256 (28%)	470 (27%)
Large Cell carcinoma	3 (0%)	14 (2%)	17 (1%)
Other	106 (13%)	103 (11%)	209 (12%)
**Smoking status, Count (%)**			
Never	46 (6%)	1 (0%)	47 (3%)
Former	301 (37%)	5 (1%)	306 (18%)
Current	156 (19%)	2 (0%)	158 (9%)
Unknown / Not provided	318 (39%)	894 (99%)	1212 (70%)
**Past history of pneumonitis, Count (%)**	43 (5%)	0 (0%)	43 (2%)
**Past history of any chronic lung disease, Count (%)**	383 (47%)	383 (42%)	766 (44%)
**Follow-up time (months) from index treatment date, Median (Q1, Q3)**	9.7 (3.7, 22.1)	10.5 (4.0, 26.6)	10.1 (3.9, 23.8)
